# Quality of mental health care among insured patients in an Indonesian psychiatric hospital

**DOI:** 10.1186/s12913-026-14805-7

**Published:** 2026-06-11

**Authors:** Rini Rachmawaty, Rr. Tutik Sri Hariyati, Muhammad Niswar, Elly Wahyudin, Agussalim Bukhari, Rati Mardatillah, Heejung Kim

**Affiliations:** 1 Department of Nursing Management, Faculty of Nursing, Hasanuddin University, Makassar, 90245 Indonesia; 2 Department of Basic Science and Fundamental of Nursing, Faculty of Nursing, Universitas Indonesia, Depok, 16424 Indonesia; 3 Department of Informatics, Faculty of Engineering, Hasanuddin University, Gowa, 92171 Indonesia; 4 Department of Pharmacy, Faculty of Pharmacy, Hasanuddin University, Makassar, 90245 Indonesia; 5 Department of Clinical Nutrition, Faculty of Medicine, Hasanuddin University, Makassar, 90245 Indonesia; 6 Nursing Management & Healthcare Quality Research Group, Hasanuddin University, Makassar, 90245 Indonesia; 7 College of Nursing and Mo-Im Kim Nursing Research Institute, Yonsei University, Seoul, South Korea

**Keywords:** Clinical pathways, Health care quality, Hospital costs, Hospital readmissions, Length of stay, Patient discharge

## Abstract

**Background:**

This study aimed to assess the quality of inpatient psychiatric care for patients covered by the National Health Insurance (NHI) programme at the Indonesian Psychiatric Hospital in Makassar.

**Methods:**

We conducted a retrospective secondary data analysis using electronic claim (e-claim) data from Indonesia’s NHI system for the 2019–2020 period, applying the Donabedian framework to evaluate the structure, process, and outcome components of psychiatric inpatient care quality. Descriptive statistical analyses including frequency distributions, cross-tabulations, and summary measures (means, standard deviations) were applied across framework dimensions. Unadjusted odds ratios (ORs) with 95% CIs and chi-square tests were conducted to evaluate associations between prolonged LOS (>42 days) and financial loss; all associations are unadjusted and descriptive.

**Results:**

Quality assessment under Indonesia’s NHI demonstrated persistent structural challenges in psychiatric inpatient care. Thirty-day readmission rates were 7.2% (113 of 1,571 unique patients) in 2019 and 5.5% (34 of 614 unique patients) in 2020, indicating gaps in continuity of care and suboptimal transition processes. Nearly half of total admissions exceeded 42 days, reflecting substantial clinical complexity and prolonged stabilisation needs. Financial evaluations showed that hospital tariffs consistently exceeded INA-CBGs reimbursement across all length of stay categories, generating universal financial losses. Prolonged stays (>42 days) accounted for approximately 80% of total loss-incurring admissions and produced the largest mean deficits, highlighting significant misalignment between the prospective payment system and the true resource requirements of psychiatric care.

**Conclusions:**

This study identifies a dual challenge in Indonesian psychiatric inpatient care: structural misalignment between INA-CBGs reimbursement and actual care costs, compounded by service-delivery factors inherent to severe and relapsing psychiatric illness. Addressing these challenges requires both tariff reform and investment in integrated clinical pathways and post-discharge community support to ensure financial sustainability and high-quality mental healthcare within Indonesia’s NHI system.

## Background

Mental health service quality is a critical priority under Indonesia’s universal health coverage scheme. Participating hospitals must ensure that care delivery adheres to six essential dimensions: safety, effectiveness, timeliness, efficiency, equity, and patient-centeredness [1–3]. However, the challenges of improving service quality in psychiatric hospitals are markedly more complex, particularly in low- and middle-income countries where healthcare resources are often constrained [4–7]. According to the Donabedian framework, healthcare quality can be assessed through three dimensions: structure (organizational characteristics), process (clinical activities), and outcomes (patient results) [8, 9]. In psychiatric settings, two key process and outcome indicators have been widely validated as proxies for care quality, length of stay (LOS) and hospital readmission rates [10–12].

Mental health disorders represent a significant global health burden, within psychiatric care settings, a critically important challenge is the ‘revolving door’ phenomenon - a pattern of frequent hospital admissions among patients with severe mental illness [13–15]. This pattern occurs when patients experience symptoms relapse shortly after discharge from hospital, often due to medication non-adherence, inadequate follow-up care, or insufficient community support systems [14, 16, 17]. The evidence from scoping reviews indicates that shorter LOS significantly correlates with increased readmission rates, primarily through diminished clinical stabilisation and reduced treatment effectiveness during the index hospitalisation [18]. As psychiatric disorders require comprehensive assessment, medication adjustment, therapeutic interventions, and structured discharge planning, insufficient LOS may result in premature discharge before achieving optimal stabilisation, consequently elevating relapse and readmission risk [19, 20]. These process deficiencies thus directly manifest as adverse outcomes, establishing the analytical pathway through which Donabedian’s process-outcome linkage operates in psychiatric inpatient care.

Following Indonesia’s Presidential Regulation No. 59 of 2024, which amended the previous Presidential Regulation No. 82 of 2018 on Health Insurance, the government continued to assign Social Security Administrative Body for Health to oversee the national health insurance (NHI) programme as part of Indonesia’s transition toward Universal Health Coverage (UHC) [21]. NHI introduced the Indonesian Case Based Groups (INA-CBGs) tariff system at the hospital level, a prospective payment model [22]. This system assigns tariffs based on diagnostic groupings that consider clinical methodologies and resource utilisation consistency. Healthcare payments are thus determined by the INA-CBGs code linked to a patient’s diagnosis, regardless of the actual treatment costs incurred.

The INA-CBGs tariff system is conceptualized as a structural determinant within the Donabedian framework, as it shapes the institutional environment in which care processes are delivered [8, 9]. When reimbursement structures are inadequate, they constrain the resources available for care delivery, ultimately compromising both process quality and patient outcomes [23]. Financial performance (the gap between actual hospital costs and INA-CBGs reimbursement) therefore functions as an outcome indicator of structural adequacy, making tariff assessment an integral component of quality evaluation rather than a purely economic concern [23]. The World Health Organization (WHO) establishes a fundamental connection between robust health financing mechanisms and the delivery of high-quality mental health services, making the assessment of INA-CBGs tariff adequacy critically important for ensuring optimal mental health care quality in Indonesia’s healthcare system [24, 25]. Therefore, the INA-CBGs reimbursement rates should be at least equivalent to, and ideally exceed, the actual treatment costs incurred by the hospital [26]. This is also supported by the findings of Mahanggi et al., (2023) which demonstrated that when INA-CBGs reimbursement rates exceed the actual treatment costs, the hospital can sustain the quality of mental healthcare services and avoid financial deficits [27]. Aligning reimbursement policies with clinical realities is imperative to ensure the long-term viability of mental healthcare services for patients with complex psychiatric needs.

While Indonesian research has explored INA-CBGs reimbursement across a range of clinical areas, such as Satibi et al. (2019), who reported that actual treatment expenditures were lower than INA-CBGs tariffs for cancer chemotherapy in a general hospital; Pratiwi et al. (2017), who examined clinical predictors of schizophrenia readmission; and Mahanggi et al. (2023), who documented cost discrepancies for schizophrenia patients in a non-specialised hospital, these studies remain condition-specific and do not address the broader financing dynamics of psychiatric inpatient care [27–29]. Critically, no study has rigorously assessed the financial sustainability or tariff adequacy of INA-CBGs within specialised psychiatric hospitals, despite these facilities caring for patient populations whose clinical trajectories differ fundamentally from those in acute medical settings [27–29].

Globally, psychiatric inpatient services are known to require longer lengths of stay and show persistently high readmission rates, placing substantial pressure on case-based reimbursement systems in many countries [19, 30]. Evidence from high-income settings shows that inadequate reimbursement for mental health admissions often leads to structural underfunding, service fragmentation, and reduced care quality issues that may be even more pronounced in low- and middle-income countries [31]. Therefore, this study aimed to assess the quality of inpatient psychiatric care for patients covered by the NHI programme at the Indonesian Psychiatric Hospital in Makassar. While extensive research has examined the relationship between LOS, readmission, and healthcare costs, evidence from Indonesian psychiatric hospitals operating under the INA-CBGs reimbursement system remains limited. Specifically, little is known about: How is the quality of psychiatric inpatient care for insured patients under the NHI scheme reflected in the distribution of LOS relative to national standards, the pattern and magnitude of hospital readmissions, and the adequacy of INA-CBGs reimbursement in relation to actual hospital costs. To operationalize this framework, each research question maps onto a distinct Donabedian dimension with specific measurable indicators: (1) Structure is assessed through patient demographics, ward-class distributions as proxies for institutional capacity and the INA-CBGs tariff system as a structural financing determinant shaping the organizational environment of care delivery; (2) Process is evaluated through LOS relative to the 42-day national standard; and (3) Outcomes are measured through 30-day readmission rates, discharge status, and financial performance (profit or loss per episode). These mappings operationalize the analytical linkage between quality dimensions and the study’s indicators.

## Methods

This study employed a secondary data analysis design to assess the quality of inpatient psychiatric care for patients covered by Indonesia’s NHI programme. The methodological approach follows the Donabedian framework for healthcare quality assessment, examining structure, process, and outcome dimensions [8, 9, 32].

### Study design and data source

This retrospective study analyzed administrative records from Indonesia’s national e-claim repository, which captures all reimbursement transactions processed through the Social Security Administration for Health (BPJS Kesehatan). The study analyzed data from two periods: January 1 to December 31, 2019, and January 1 to July 31, 2020. Indonesia confirmed its first COVID-19 cases in March 2020, and subsequent public health measures may have influenced psychiatric service utilisation patterns during the 2020 study period. The comparison between 2019 and 2020 data therefore reflects both routine variation and potential early pandemic effects on hospital admissions, length of stay, and readmission rates.

### Setting and sample

The study was conducted at a government-operated psychiatric hospital in Makassar, South Sulawesi Province, Indonesia. This facility serves as the primary referral centre for psychiatric care in the eastern Indonesia region, with a total bed capacity of 363 beds distributed across first-class (private), second-class (semi-private), and third-class (general) inpatient wards. As the sole tertiary psychiatric referral centre for the eastern Indonesia region, this hospital manages a disproportionately complex case-mix relative to community psychiatric health centres, district-level psychiatric units, and secondary psychiatric hospitals. Consequently, the clinical complexity of the patient population, the LOS distribution, and the magnitude of INA-CBGs reimbursement deficits observed in this study may not be representative of other Indonesian psychiatric facilities operating at lower levels of care. Potential institutional-level selection bias should be considered when interpreting these findings in the broader national context.

Study variables were organised according to the Donabedian quality framework, categorized into structure, process, and outcomes. Regarding participant selection, the study included all patients enrolled in the NHI programme who were admitted to inpatient psychiatric units and formally discharged within the designated study periods. Patients who were not covered under the NHI scheme (including those under private pay or alternative insurance arrangements), those with incomplete admission records, and cases involving transfer to another facility prior to discharge were excluded from the analysis.

### Study measures

Study variables were organised according to the Donabedian quality framework, categorized into structure, process, and outcome dimensions. The structure category included patient demographics such as gender, age, inpatient ward classification (1st, 2nd, or 3rd class), and the nature of the hospital admission (initial or readmission). The process category examined diagnoses based on the Indonesian Case-Based Groups (INA-CBGs) and categorized the LOS as either less than or more than 42 days. The outcomes category assessed discharge status (physician approval, referral, patient request, death, or other reasons), hospital financial performance (profit or loss), and 30-day readmission rates (whether patients were readmitted once, twice, three times, or four times).

#### INA-CBGs reimbursement system

Indonesia’s NHI programme Jaminan Kesehatan Nasional (JKN), administered by the BPJS Kesehatan reimburses secondary and tertiary care facilities through the Indonesian Case-Based Groups (INA-CBGs) framework, a prospective case-mix payment system established under Ministry of Health Regulation No. 52 of 2016. Under this mechanism, hospitals are compensated through fixed bundled payments determined by groupings of disease diagnoses and medical procedures, irrespective of the actual costs incurred during the episode of care. The INA-CBGs tariff encompasses all medical and non-medical resource utilisation within a single payment package, thereby creating incentives for cost containment and clinical efficiency at the provider level [33].

#### Length of stay classification

Length of stay was dichotomized using the 42-day threshold established in Indonesian psychiatric care standards. This benchmark aligns with the national regulatory framework outlined in the Decree of the Minister of Health No. 129/Menkes/SK/II/2008 on Hospital Minimum Service Standards and the Regulation of the Minister of Health No. 741/2008 [34]. Both regulations identify 42 days as the maximum acceptable duration for effective inpatient psychiatric treatment, reflecting the clinically appropriate period required for stabilisation, medication adjustment, and discharge planning in patients with severe mental disorders. Hospitalisations exceeding 42 days were therefore classified as “prolonged stays,” indicating complex clinical presentations, treatment resistance, or the need for extended therapeutic interventions.

#### Hospital readmission

Hospital readmission was defined as any subsequent admission to the same facility within 30 days of discharge from the index hospitalisation, consistent with internationally accepted quality metrics for psychiatric care. Readmission episodes were further categorized as first, second, or third readmissions to capture the ‘revolving door’ phenomenon characteristic of severe psychiatric illness.

#### Financial performance

To assess hospital financial performance, we compared the actual treatment costs documented in institutional billing records (hereafter referred to as hospital costs) against the corresponding INA-CBGs reimbursement tariffs. Admissions in which hospital costs exceeded the reimbursement amount were categorized as loss-generating cases, whereas those in which reimbursement equalled or surpassed hospital costs were categorized as profit-generating cases. The magnitude of financial loss was determined by computing the difference between hospital costs and the INA-CBGs reimbursement. Hospital costs were derived from the institution’s official cost-centre accounting system, which records all itemized treatment expenditures per admission, encompassing eight cost categories: consultation fees, nursing services, radiology services, laboratory services, rehabilitation, intensive care, medication costs, and healthcare devices. These figures were cross-referenced against the nationally published BPJS Kesehatan INA-CBGs tariff schedule applicable to the 2019–2020 period to verify consistency with the reimbursement amounts claimed. Although no independent external audit was conducted, the cost-centre data are subject to regulatory compliance review through the claim submission process, and the directional consistency of findings across both years provides additional internal validity evidence.

### Ethical considerations

Ethical approval for the study was obtained from the Research Ethics Committee for Health Sciences Research, Faculty of Nursing, Hasanuddin University (Number 1527/UN4.18.3/TP.01.02/2024), prior to accessing the NHI e-claim database for data collection.

### Data analysis

Descriptive statistics were computed for all study variables. Continuous variables (age, length of stay, hospital costs, INA-CBGs tariffs, financial differences) were summarized using means (M), standard deviations (SD), medians, and minimum and maximum values. Categorical variables (gender, ward type, admission category, discharge status, LOS category, financial outcome, readmission status) were described using frequencies (n) and percentages (%).

The analysis was structured according to the Donabedian framework dimensions:

1. **Structure assessment:** The distribution of patient demographics (age, gender), ward classifications, and admission categories was examined using frequency distributions and percentages.
2. **Process assessment:** The distribution of length of stay was analyzed by categorizing admissions into ≤42 days and >42 days groups. INA-CBGs diagnostic codes were evaluated to identify high-volume, high-risk, and high-cost diagnoses.
3. **Outcome assessment:** Financial performance was assessed by calculating the proportion of admissions resulting in profit versus loss. For loss-generating admissions, the distribution of financial losses was examined across LOS categories (≤42 days vs. >42 days) to identify patterns. A cost component analysis was conducted to identify the primary drivers of financial losses. Hospital readmission patterns were analyzed by examining 30-day readmission rates, readmission episodes, and their relationship with discharge status and LOS categories.

Cross-tabulations were used to examine the distribution of key variables across categories, including financial outcomes by LOS category, readmission episodes by discharge status, and readmission episodes by LOS category. Unadjusted odds ratios (ORs) with 95% confidence intervals (CIs) were calculated for the association between prolonged LOS (>42 days) and financial loss, and chi-square tests were conducted to formally evaluate these associations. Mean differences in financial loss by LOS category with 95% CIs were calculated using independent-samples t-tests. All reported associations are unadjusted and descriptive; adjusted multivariable analyses were not conducted, as the administrative e-claim dataset does not include validated clinical severity instruments (e.g., BPRS, PANSS), which represent the most consequential confounders in predictive models of psychiatric readmission and financial outcome. For inferential analyses, an index-admission approach was adopted (retaining only the first admission per patient within each study period) to mitigate within-patient clustering; sensitivity analyses treating all admissions as independent observations confirmed directional consistency. Future research should apply cluster-robust standard errors, generalized estimating equations (GEE), or mixed-effects models to formally account for repeated-measures dependency in these data. Data were analyzed separately for 2019 and 2020 to allow for temporal comparison while accounting for differences in the observation periods. All analyses were performed using IBM SPSS Statistics version 26.0 (IBM Corp., Armonk, NY, USA).

## Results

**Fig. 1:**
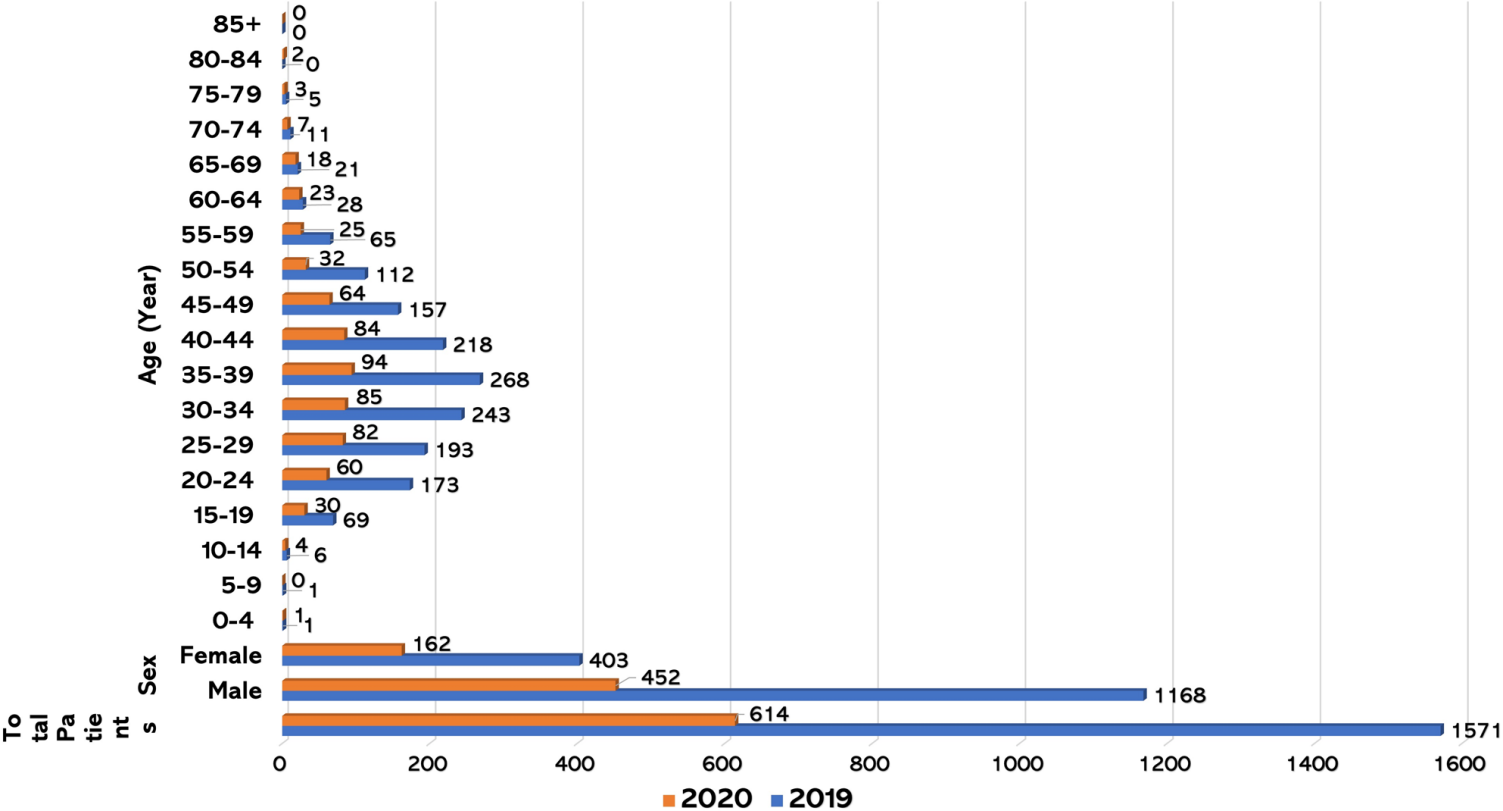
Distribution of total patients hospitalised in Indonesian psychiatric hospital by age and sex in 2019 and 2020

Figure 1 shows the results of the analysis of the distribution of total patients hospitalised in the Indonesian psychiatric hospital located in Makassar city, South Sulawesi Province, during the 2019–2020 period and covered by the NHI; it was found that the total number of patients in inpatient wards were 1,571 in 2019 and 614 in 2020. The significant difference in the number of patients in these two periods is due to the e-claim data obtained for the 2020 period is inpatient data that has a discharge date only from January 1 – July 31, 2020; and are dominated by male patients. Gender distribution analysis reveals that male patients represented approximately 74.3% of total patients in 2019 and 73.6% in 2020, highlighting a consistent gender disparity in psychiatric hospitalisation. Most hospitalized patients in 2019 were aged 35–39 years (17%), meanwhile, in 2020, most patients were aged 35–39 years (15.3%).

**Fig. 2:**
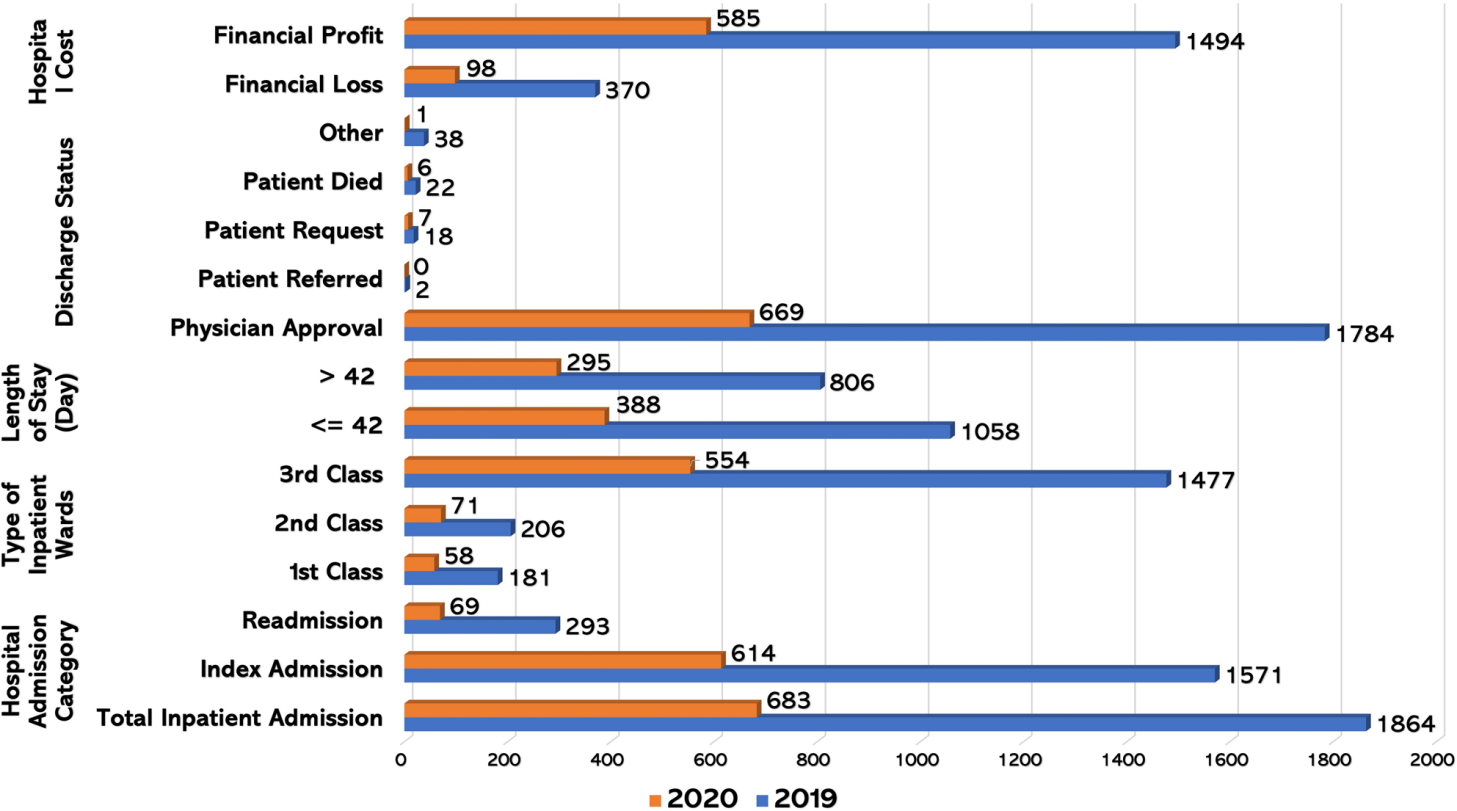
Distribution of total inpatient admissions, hospital admission categories, types of inpatient wards, length of hospital stays, discharge status, and hospital costs during 2019 (*n* = 1,864) and 2020 (*n* = 683) in the Indonesian psychiatric hospital

Figure 2 presents the distribution of inpatient admissions by admission category, ward type, length of stay, discharge status, and financial outcome at the study hospital during 2019–2020. Total admissions in 2019 reached 1,864 admissions, while 683 admissions were recorded in 2020. Readmissions accounted for 15.7% (293 admissions) of total admissions in 2019 and 10% (69 admissions) of total admissions in 2020, reflecting the hospital’s approach to patient care. Analysis of LOS patterns shows that in 2019, short stays (≤42 days) accounted for the majority of inpatient episodes, representing 1,058 of 1,864 admissions (56.7%), while prolonged stays (>42 days) comprised 806 admissions (43.3%). In 2020, this distribution remained similar, with 388 of 683 admissions (56.8%) falling within the ≤42-day category, and 295 admissions (43.2%) exceeding 42 days. Financial indicators highlight a persistent misalignment between service delivery costs and national insurance reimbursement. The hospital incurred significant financial losses, with 370 loss-generating admissions in 2019 and 98 in 2020. Although the total number decreased in 2020, the proportion relative to total admissions remained notable. These trends point to structural limitations in the INA-CBGs tariff system for psychiatric care, particularly for complex or prolonged admissions that often require resource-intensive management (Fig. 2).

**Fig. 3:**
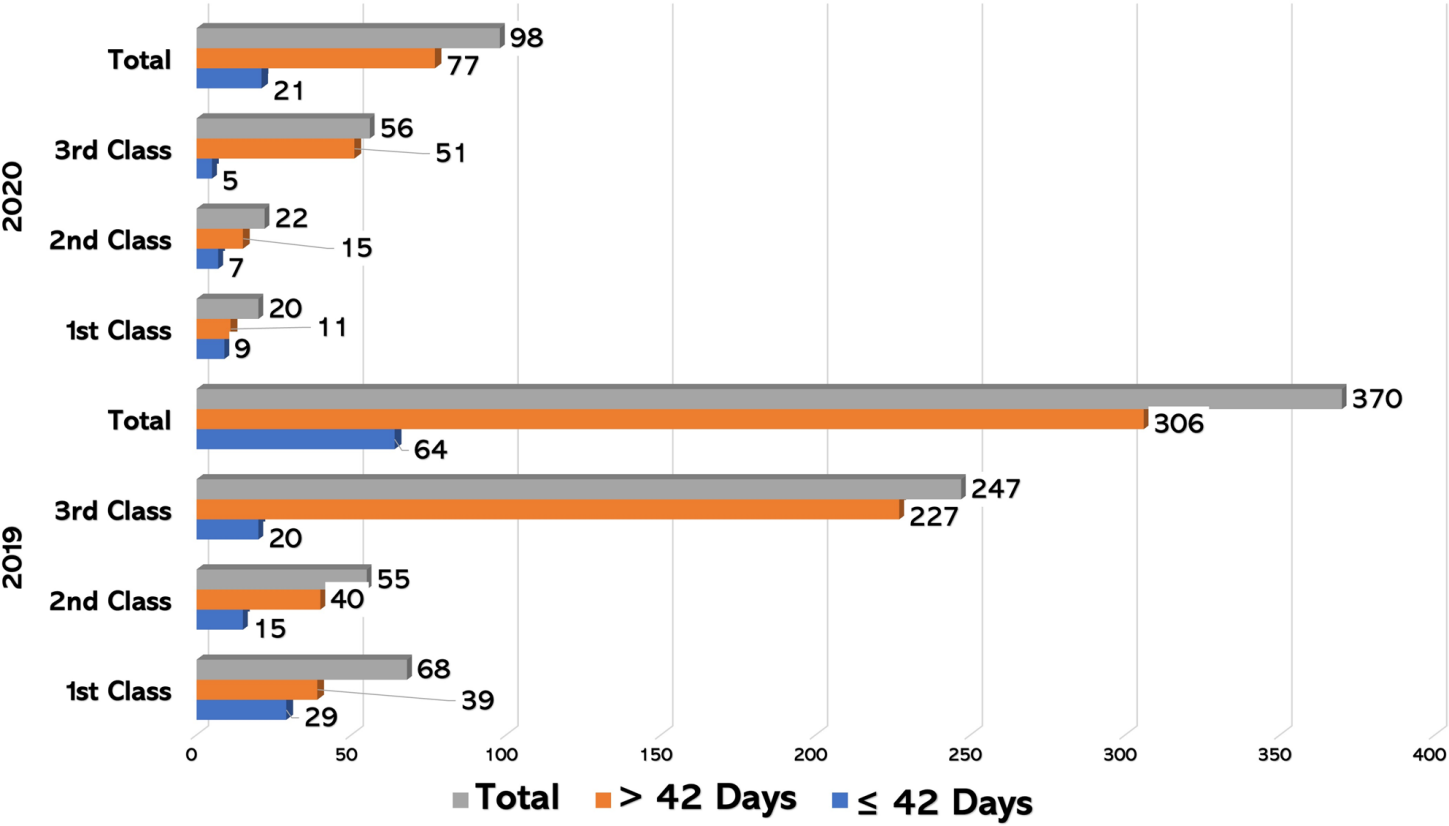
Distribution of total inpatient admissions causing financial loss by types of inpatient wards & LOS in the Indonesian psychiatric hospital 2019 (*n* = 370) and 2020 (*n* = 98)

Figure 3 shows that analysis of inpatient admissions associated with financial losses, prolonged stays (>42 days) accounted for the majority in both years. In 2019, among the 370 admissions that generated financial loss, 306 cases (82.7%) were prolonged stays **(>**42 days), whereas only 64 cases (17.3%) were shorter stays (≤42 days). In 2020, a similar pattern was observed of the 98 loss-incurring admissions, 77 cases (78.6%) were prolonged stays **(>**42 days), while 21 cases (21.4%) were shorter stays (≤42 days) (Fig. 3). These findings demonstrate that prolonged stays **(>**42 days) consistently accounted for the largest proportion of financial losses, highlighting the substantial economic burden associated with extended psychiatric hospitalisations.

**Fig. 4:**
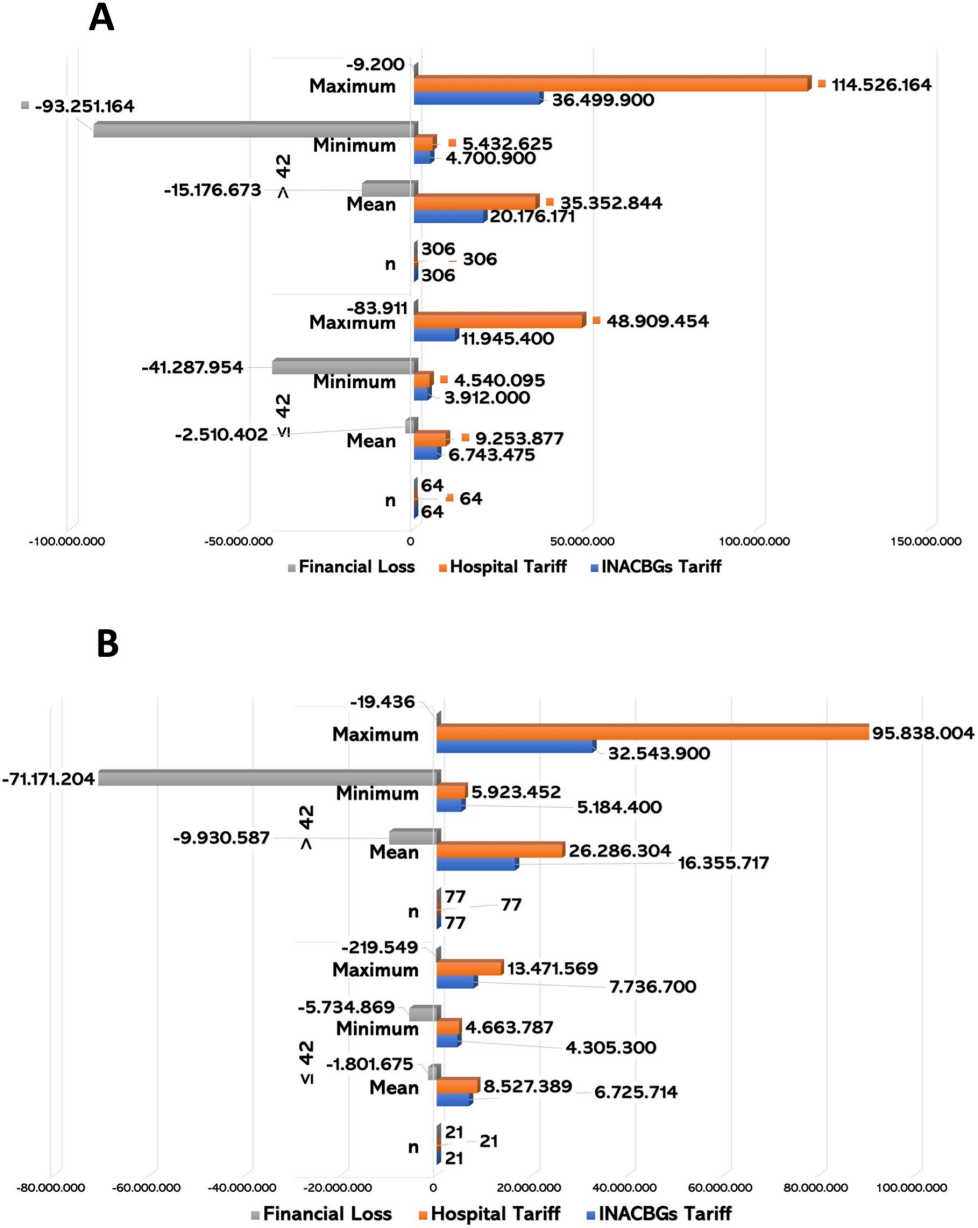
Distribution of INA-CBGs tariff, hospital tariff & financial loss of insured patients hospitalized in the Indonesian psychiatric hospital in 2019 (*n* = 370) and 2020 (*n* = 98)

The distribution of INA-CBGs Tariff, Hospital Tariff, and Financial Loss found in Insured Patients Hospitalized at Indonesian psychiatric hospital. In 2019, a substantial mismatch between hospital tariffs and INA-CBGs reimbursement resulted in significant financial losses for the psychiatric hospital. Patients with prolonged stays (>42 days) experienced an average loss of IDR 15,176,673, with individual losses reaching up to IDR 93,251,164. Even among patients with shorter stays (≤42 days), the hospital still incurred an average loss of IDR 2,510,402, and some admissions recorded losses as high as IDR 41,287,954 (Fig. 4A). A similar pattern persisted in 2020, during which patients with prolonged stays (>42 days) included admissions that incurred financial losses reaching IDR 71,171,204, whereas among those with shorter stays (≤42 days) there were admissions with losses of up to IDR 5,734,869, highlighting the continued financial burden imposed by the INA-CBGs reimbursement structure (Fig. 4B).

Across both years, a consistent pattern emerged that hospital tariff exceeded INA-CBGs reimbursement across all LOS categories, resulting in universal financial losses. Prolonged stays (>42 days) incurred dramatically higher mean losses compared with shorter stays and were responsible for the largest financial deficits each year. The magnitude of these losses demonstrates a misalignment between the INA-CBGs prospective payment model and the actual resource demands of psychiatric inpatient care, particularly for chronic or severe cases requiring extended hospitalisation.

Unadjusted analysis confirmed a strong and consistent association between prolonged hospitalisation and financial loss across both study years. In 2019, prolonged-stay admissions (>42 days; *n* = 806) had a financial loss rate of 37.9% (306/806), compared with 6.0% (64/1,058) among shorter-stay admissions (≤42 days), yielding substantially higher odds of loss [OR = 9.51; 95% CI: 7.11–12.70; χ^2^ (1) = 292.91, *p* < .001; *N* = 1,864]. In 2020, a consistent pattern was observed, with prolonged-stay admissions (*n* = 295) demonstrating a financial loss rate of 26.1% (77/295) compared with 5.4% (21/388) for shorter stays [OR = 6.17; 95% CI: 3.70–10.29; χ^2^ (1) = 58.37, *p* < .001; *N* = 683]. Across both years, the 95% confidence intervals excluded unity, confirming that the associations are statistically significant. The attenuated OR in 2020 relative to 2019 (6.17 vs. 9.51) is consistent with the reduced sample size and altered admission patterns attributable to early COVID-19 pandemic disruptions during the January–July 2020 study period, rather than a genuine improvement in reimbursement adequacy, as the financial loss rate among shorter-stay admissions remained stable across both years (6.0% vs. 5.4%). The absolute magnitude of financial losses also differed substantially between LOS categories. In 2019, the mean financial loss per prolonged-stay admission was IDR 15,176,673 (SD = IDR 15,702,971), compared with IDR 2,510,402 (SD = IDR 5,175,618) among shorter-stay admissions; the mean difference was IDR 12,666,270 (95% CI: IDR 10,488,911–IDR 14,843,630) [t(305.33) = 11.45, *p* < .001]. In 2020, mean financial loss per prolonged-stay admission was IDR 9,930,587 (SD = IDR 15,316,776) versus IDR 1,801,675 (SD = IDR 1,595,068) for shorter stays, yielding a mean difference of IDR 8,128,912 (95% CI: IDR 4,587,962–IDR 11,669,862) [t(81.67) = 4.57, *p* < 0.001]. As Levene’s test indicated unequal variances between LOS groups in both years (2019: F = 68.46, *p* < .001; 2020: F = 12.99, *p* < .001), Welch’s t-test was applied to all mean comparisons. All reported estimates are unadjusted and reflect descriptive associations; the absence of validated clinical severity instruments (e.g., BPRS, PANSS) in the administrative e-claim dataset precluded adjustment for illness severity, the most consequential unmeasured confounder in models predicting psychiatric financial outcomes, and findings should be interpreted accordingly.

**Fig. 5:**
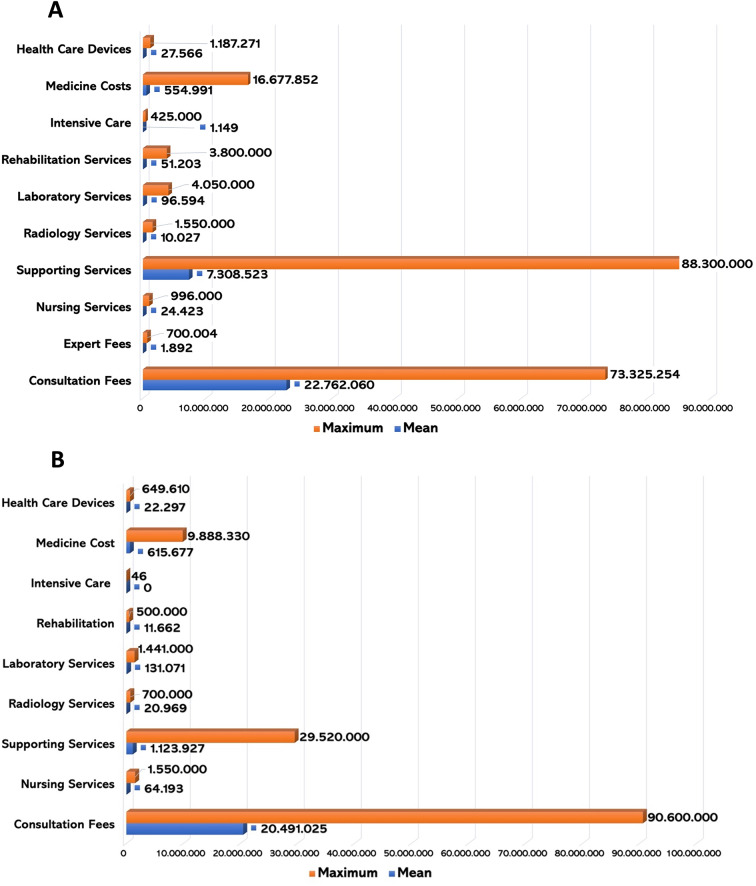
Breakdown of hospital costs that causing financial loss based on insured patients hospitalized in the Indonesian psychiatric hospital in 2019 (*n* = 370) and 2020 (*n* = 98)

Cost component analysis (Fig. 5). The data from 2019 and 2020 reveal that the three primary factors driving financial losses were medication costs, supporting services, and consultation fees. These financial deficits can negatively impact the hospital’s ability to access capital and may lead to higher borrowing costs, further straining its financial health [35]. Given that enhancing hospital quality and patient safety often demands substantial investment, facilities facing financial difficulties due to inadequate revenue might reduce their quality improvement initiatives, exacerbating the situation [36]. To assess the scale of these losses, we identified and analyzed the top 10 INA-CBGs diagnoses responsible for the highest costs.

Indonesia is one of the countries with significant mental health problems. Based on the calculation of disease burden in 2017, Indonesia has experienced several types of mental disorders, including depression, anxiety, schizophrenia, bipolar disorder, behavioural disorders, autism, eating behaviour disorder, intellectual disability, and Attention Deficit Hyperactivity Disorder (ADHD) [37, 38]. Analysis of the INA-CBGs diagnostic categories presented in Table 1 demonstrates that schizophrenia-spectrum disorders dominate the caseload and are the primary contributors to financial losses in both 2019 and 2020. Across both years, diagnoses such as F20.9 (Schizophrenia, unspecified), F20.0 (Paranoid schizophrenia), and F25.0 (Schizoaffective disorder, bipolar type) consistently appear as *high-volume* conditions indicating that they account for the largest proportion of psychiatric inpatient admissions.

**Table 1:** 10 INA-CBGs diagnoses categorized as high volume, high risk, & high-cost that causing financial loss during the patient hospitalisation in Indonesian psychiatric hospital during 2019 and 2020

2019 (n = 370)				2020 (n = 98)			
INA-CBGs Diagnoses	Length of Stay (LOS)		Total	INA-CBGs Diagnoses	Length of Stay (LOS)		Total
	≤ 42 Days	> 42 Days			≤ 42 Days	> 42 Days	
F20.9 (Schizophrenia, unspecified)	27	208	235	F20.9 (Schizophrenia, unspecified)	12	41	53
F20.0 (Paranoid schizophrenia)	7	18	25	F20.0 (Paranoid schizophrenia)	4	6	10
F25.0 (schizoaffective disorder, bipolar type)	8	13	21	F25.0 (schizoaffective disorder, bipolar type)	1	6	7
F29 (Unspecified psychosis not due to a substance or known physiological condition)	2	15	17	F29 (Unspecified psychosis not due to a substance or known physiological condition)	0	3	3
F06.8 (Other specified mental disorders due to known physiological condition)	1	5	6	F31.2 (bipolar disorder, current episode manic severe with psychotic features)	1	1	2
F20.1 (Disorganized schizophrenia)	3	2	5	F06.9; S01.9 (Unspecified mental disorder due to brain damage and dysfunction and to physical disease; Open wound of unspecified part of head)	0	1	1
F20.5 (Residual schizophrenia)	1	2	3	F10.0 (Mental and behavioural disorders due to use of alcohol, acute intoxication)	0	1	1
F31.2 (bipolar disorder, current episode manic severe with psychotic features)	1	2	3	F20.0; L02.9; L28.2;L30.0 (Paranoid schizophrenia; Cutaneous abscess, furuncle and carbuncle, unspecified; Other prurigo; Nummular dermatitis)	0	1	1
F06.9 (Unspecified mental disorder due to brain damage and dysfunction and to physical disease)	2	0	2	F20.9; E11.5 (Schizophrenia, unspecified; Type 2 diabetes mellitus with circulatory complications)	0	1	1
F20.9; A01.0 (Schizophrenia, unspecified; Typhoid fever)	1	1	2	F20.9; E87.6 (Schizophrenia, unspecified; Hypokalemia)	0	1	1

**Fig. 6:**
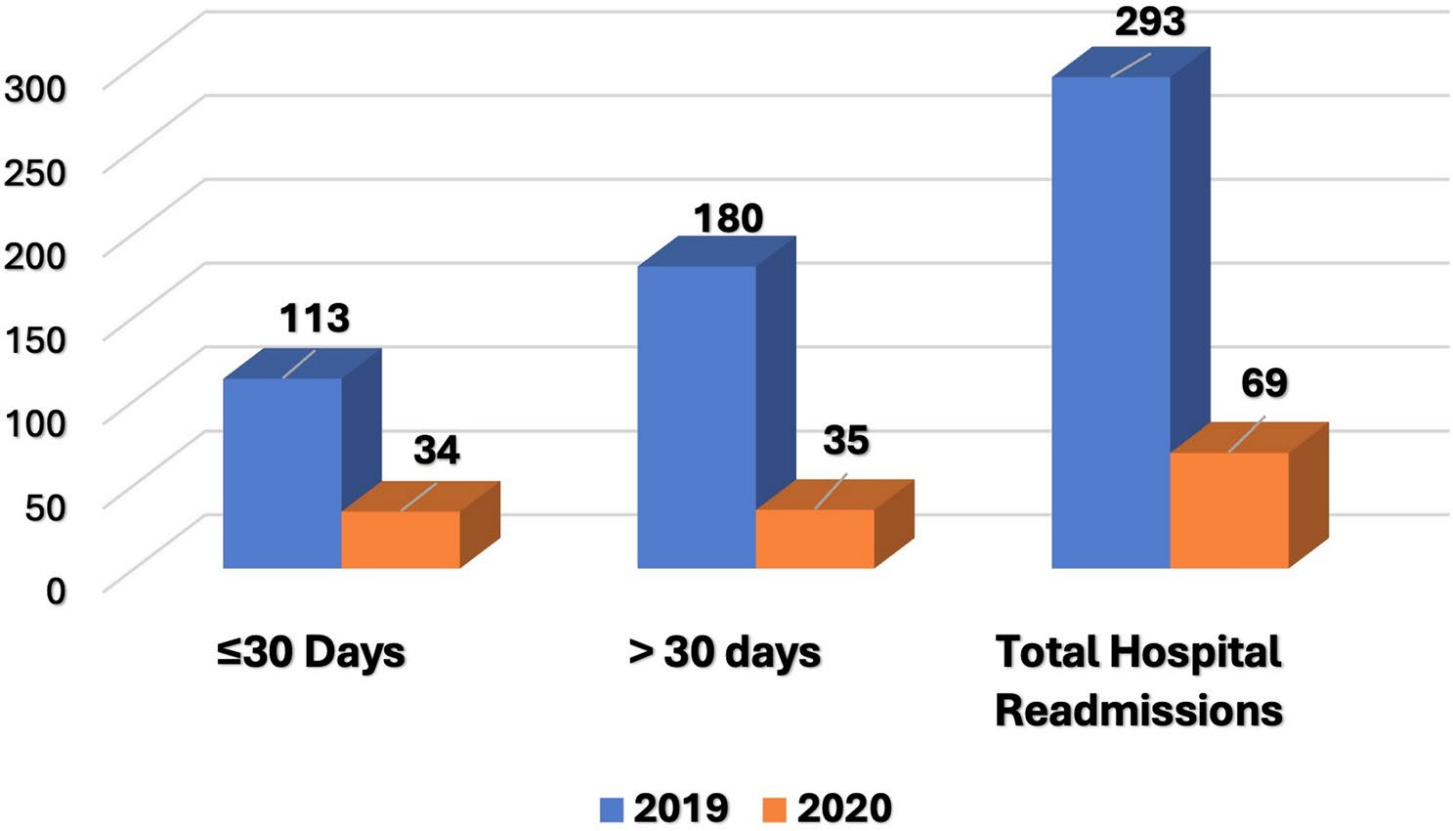
Hospital readmission status of insured patients hospitalized in the Indonesian psychiatric hospital in 2019 & 2020

Based on Fig. 6 we can conclude that the total hospital readmission of insured patients had a fairly high number in 2019, which was 293 cases, of which in 2020 it was 69 cases. Thirty-day hospital readmissions are an indicator of quality of care; hospitals are financially penalised by Medicare for high rates. Numerous care transition processes reduce readmissions in clinical trials [39]. Hospital readmission where this is an indicator of quality as well as a measure of assessment in terms of hospital services and management. Besides its use as a measure of quality, readmissions can have financial consequences for hospitals [40]. Several studies have examined the effectiveness of care pathways; however, a review of the quality of these studies demonstrates poor overall scientific quality [41, 42]. Specifically, several studies are flawed by use of historical controls without adequate adjustment for secular trends. Comprehensive reviews of care pathways in general medical and surgical settings also show weak evidence for reducing length of stay or improving care [38, 41, 43].

**Fig. 7:**
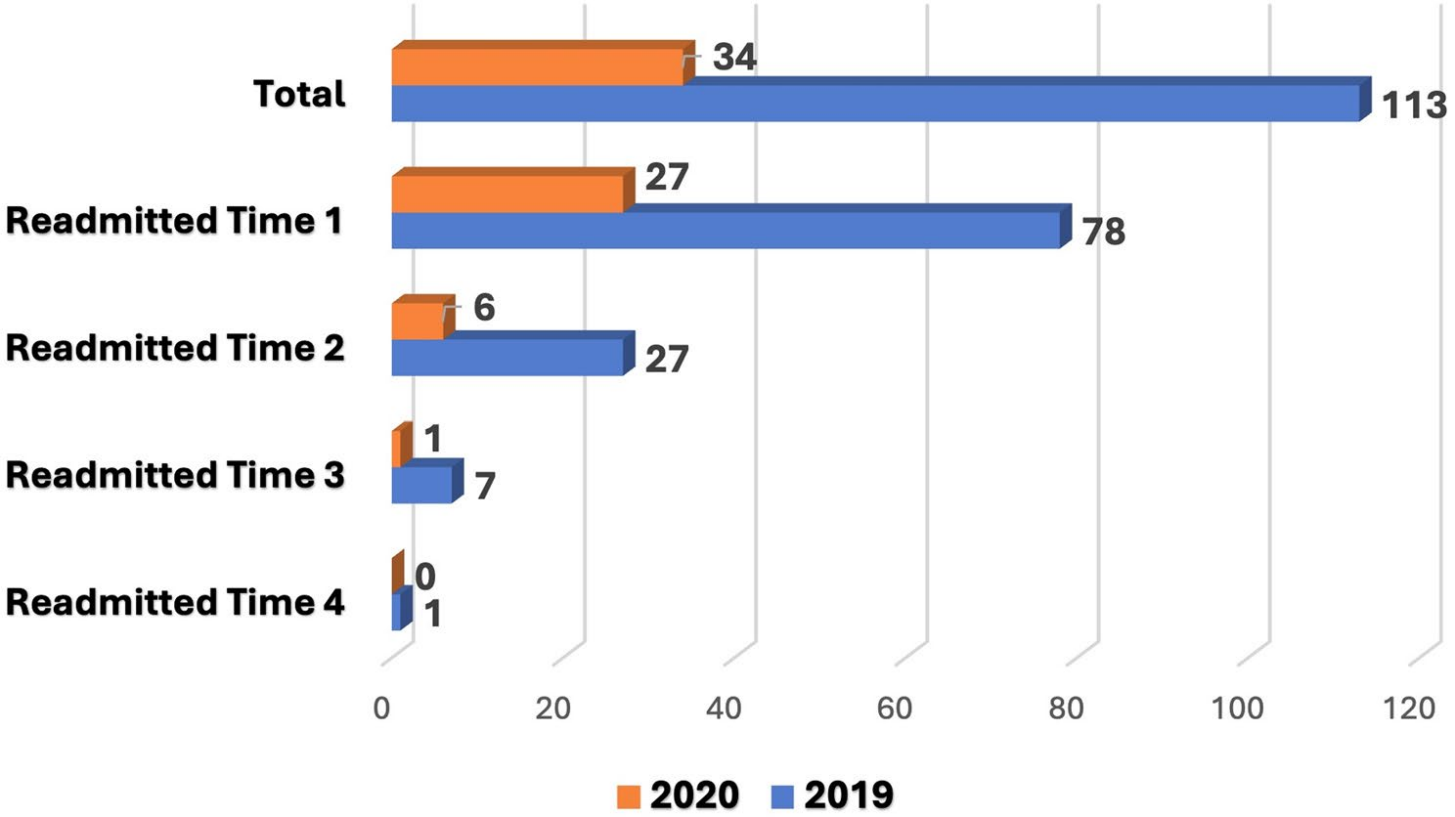
Breakdown of 30-days hospital readmissions in Indonesian psychiatric hospital in 2019 and 2020

From Fig. 7 it is known that the total number of readmission patients in Indonesia in 2019 was 113 and as many as 34 in 2020. Readmission in time 1 had the highest number in 2019 at 78 cases. While the extent to which readmissions are preventable is still widely debated [44]. and hospitals have generally focused attention on improving care transitions as a strategy to reduce preventable readmissions [45]. But Burke found that the more domains of the ideal transitions in care framework were addressed in an intervention, the more likely it was to significantly reduce readmissions [46].

Based on the analysis of both tables (Tables 2 & 3), it was found that even patients with prolonged stays (>42 days) still showed readmission rates within 30 days. In 2019, readmitted time 1 with physician approval status showed that 33 out of 113 patients (29.2%) had prolonged stays (>42 days), and readmitted time 2 showed 14 patients (12.3%) had prolonged stays (>42 days). A similar pattern was also observed in 2020, where readmitted time 1 with physician approval status showed 10 out of 34 patients (29.4%) had prolonged stays (>42 days), and readmitted time 2 showed 4 patients (11.7%) had prolonged stays (>42 days).

**Table 2:** Distribution of LOS of insured patients who experienced with ≤30 Days readmission rates by discharge status & readmission episodes in Indonesian psychiatric hospital in 2019 (*n* = 113)

Hospital Readmission Episodes	Discharge Status	Length of Hospital Stays (Days) 2019		
		≤42 Days (*n* = 61)	>42 Days (*n* = 52)	Total (*n* = 113)
Readmitted Time 1	Physician Approval	37	33	70
	Patient Request	1	0	1
	Patient Died	0	3	3
	Other	2	2	4
	Sub Total	40	38	78
Readmitted Time 2	Physician Approval	13	14	27
Readmitted Time 3	Physician Approval	7	0	7
Readmitted Time 4	Physician Approval	1	0	1
Grand Total		61	52	113

**Table 3:** Distribution of LOS of insured patients who experienced with ≤30 Days readmission rates by discharge status & readmission episodes in Indonesian psychiatric hospital in 2020 (*n* = 34)

Hospital Readmission Episodes	Discharge Status	Length of Hospital Stays (Days) 2020		
		≤42 Days (n = 18)	>42 Days (n = 16)	Total (n = 34)
Readmitted Time 1	Physician Approval	15	10	25
	Patient Request	0	1	1
	Other	0	1	1
	**Sub Total**	**15**	**12**	**27**
Readmitted Time 2	Physician Approval	2	4	6
Readmitted Time 3	Physician Approval	1	0	1
Grand Total		**18**	**16**	**34**

Notably, the overwhelming majority of 30-day readmissions occurred among patients discharged with physician approval. In 2019, physician-approved discharges accounted for 92.9% of all 30-day readmissions (105/113), with the remaining 7.1% (8/113) comprising patient-request discharges (*n* = 1), deaths (*n* = 3), and other discharge categories (*n* = 4). In 2020, physician-approved discharges similarly accounted for 94.1% of all 30-day readmissions (32/34), with only 2 cases (5.9%) discharged under other circumstances. This pattern strongly suggests that readmission risk was not concentrated among patients prematurely discharged against medical advice, but rather reflects the natural relapsing and chronic course of severe psychiatric illness particularly schizophrenia-spectrum disorders following clinically appropriate discharge. These findings underscore the importance of robust post-discharge community support and structured follow-up care in reducing preventable readmissions, irrespective of discharge status.

## Discussion

This study assessed the quality of psychiatric inpatient care for patients covered by Indonesia’s NHI programme, examining three key dimensions: LOS distribution, hospital readmission patterns, and financial adequacy of INA-CBGs reimbursement. The findings reveal significant structural challenges within the current healthcare financing system for psychiatric services, with particular impact on clinical process indicators and institutional financial performance.

### Length of hospital stay distribution and readmission patterns

Our findings demonstrate that a substantial proportion of psychiatric hospitalisation exceeded the 42-day benchmark, with 43.3% in 2019 and 43.2% in 2020 requiring prolonged care. This pattern reflects the inherent clinical complexity of severe mental disorders and aligns with international evidence demonstrating that psychiatric conditions require comprehensive assessment, medication optimization, therapeutic interventions, and structured discharge planning that cannot be adequately accomplished within shortened timeframes [19, 20].

The predominance of schizophrenia-spectrum disorders (F20.9, F20.0) and schizoaffective disorder (F25.0) among high-volume, high-risk, and high-cost diagnoses explains the extended hospitalisation requirements. International literature confirms that patients with schizophrenia have mortality rates two to three times higher than the general population and require intensive, resource-demanding treatment approaches [47]. Schizophrenia consistently emerges as the most frequently hospitalized psychiatric diagnosis, generating the highest costs and contributing substantially to prolonged lengths of stay [48]. The chronic and relapsing nature of these conditions necessitates longer stabilisation periods to achieve therapeutic goals and reduce subsequent readmission risk.

The relationship between LOS and readmission risk demonstrates a dose-response pattern consistent with international evidence. Research indicates that patients are 3.52 times more likely to be readmitted with short inpatient stays (LOS ≤ 7 days), 3.20 times with medium stays (LOS 8–31 days), and 1.91 times with extended stays (LOS 32–92 days) compared to patients treated for more than 92 days [49, 50]. This graduated risk reduction underscores the protective effect of adequate hospitalisation duration, yet conflicts directly with the financial pressures imposed by prospective payment systems.

From the Donabedian framework perspective, our LOS findings reflect both process and outcome dimensions of care quality. Adequate LOS represents a critical process measure ensuring sufficient time for clinical stabilisation, while premature discharge driven by financial constraints has been associated with compromised patient outcomes in the psychiatric literature [8, 9]. Meta-analytic evidence demonstrates that shorter LOS significantly correlates with increased readmission rates through diminished clinical stabilisation and reduced treatment effectiveness during index hospitalisation [18].

However, this pattern stands in contrast to evidence from health-service research demonstrating that reductions in LOS are achievable not by accelerating discharge, but through targeted improvements in service delivery. Studies have shown that strengthening continuity of care, minimizing ward transfers, ensuring consistent clinical leadership, and implementing structured short-stay assessment units can significantly reduce LOS without compromising clinical outcomes [51]. Consistent with this, the present data show that 30-day readmissions occurred even among patients with prolonged hospitalisation (>42 days), in both 2019 and 2020, indicating that extended inpatient stays do not automatically confer sufficient clinical stabilisation to prevent relapse. Therefore, strengthening service coordination through the implementation of a clinical pathway becomes essential to ensure a more standardized, effective, and readmission-preventive care process [52].

Although both the absolute number and the proportion of readmissions declined in 2020 compared with 2019, the readmission rate remained clinically significant despite a substantial reduction in total inpatient admissions. This pattern may be partially explained by the early disruptions caused by the onset of the COVID-19 pandemic, which limited access to routine outpatient services, reduced community-based follow-up, and weakened psychosocial support systems [53, 54]. Such disruptions may have compromised continuity of care, thereby maintaining a persistent risk of early relapse and readmission even as hospitalisation volumes decreased.

### Financial adequacy of INA-CBGs reimbursement

The financial analysis reveals a fundamental misalignment between the INA-CBGs prospective payment model and the actual resource requirements of psychiatric inpatient care. Hospital tariffs consistently exceeded INA-CBGs reimbursement across all LOS categories, generating universal financial losses. This structural deficit has meaningful implications for healthcare quality, as hospitals facing persistent financial shortfalls may be compelled to reduce quality improvement initiatives, limit investment in staff training, or restrict access to essential medications and therapeutic services [36, 55]. Cost component analysis identified medication expenses, supporting services (including dietary and laundry), and consultation fees as the primary drivers of financial losses.

This study’s findings align with global evidence indicating that prospective payment mechanisms based on the Diagnosis Related Group (DRG) classification, although originally designed to enhance the efficiency, optimize resource allocation, and control healthcare cost, face significant limitations when applied to clinically complex and resource-intensive patient groups. A systematic review by Barouni et al. (2020) reported that prospective mechanisms based on the DRG-based system frequently encounter rising expenditures, especially among patients with severe conditions and those requiring specialised care [55]. Further, a comprehensive knowledge synthesis by Ren et al. examining 297 DRG practice studies worldwide found that psychiatric illness demonstrated the poorest cost predictability among all medical specialties [56].

Consistent with global trends, several high-income countries within the Organization for Economic Co-operation and Development (OECD) have begun reforming DRG system due to their limitations in financing complex and long-stay cases. Reforms include shifting from full DRG-based payment to blended models combining DRGs with global budgets, introducing add-on payments for complex care, implementing episode-based or bundled payments, and offering targeted financial protections for rural or specialised hospitals [56]. These reforms acknowledge that prospective payment systems require adaptation to account for substantial variation in clinical complexity and resource intensity, particularly in psychiatric care.

Within this study, the distribution of financial losses demonstrates a clear and consistent pattern. Prolonged hospitalisations (>42 days) were associated with the majority of loss-generating admissions 82.7% in 2019 and 78.6% in 2020. Losses reached an average of IDR 15,176,673 per prolonged-stay patient, with some cases incurring deficits up to IDR 93,251,164. Even short-stay patients generated average losses of IDR 2,510,402, indicating that reimbursement inadequacies affect all patient groups, though disproportionately impacting complex cases requiring extended care. Taken together, these findings **s**uggest preliminary empirical evidence that the current INA-CBGs model insufficiently captures the clinical heterogeneity, resource intensity, and prolonged recovery trajectories characteristic of psychiatric inpatient treatment, and therefore may warrant structural reassessment in line with global DRG reform trends. These associative findings should be interpreted with caution. Financial outcomes in hospital settings are multifactorial and may be influenced by factors beyond LOS alone, including INA-CBGs diagnostic coding accuracy and completeness, local case-mix composition and comorbidity burden, institutional capacity for discharge planning and case management, workforce staffing ratios and medication formulary access, and broader policy-level factors such as tariff revision cycles. The OR estimates and mean loss differentials (2019: OR = 9.51; 2020: OR = 6.17) provide descriptive quantification of this consistent pattern across both study years; however, as all estimates are unadjusted and derived from a single tertiary referral centre, they represent preliminary, hypothesis-forming observations rather than definitive causal evidence. Multi-centre replication incorporating validated clinical severity data is required before systemic causal attributions can be established or these findings used to inform national policy.

### Demographic patterns

The pronounced male predominance in psychiatric hospitalisations (74.3% in 2019 and 73.6% in 2020) reflects both biological and sociocultural factors specific to the Indonesian context. From a clinical perspective, men tend to experience earlier onset of severe mental disorders, more pronounced symptoms, and poorer social functioning and impulse control, making them more vulnerable to disorganised behaviour requiring hospitalisation [57, 58]. Additionally, male patients more frequently present with psychiatric comorbidities, substance use histories, and familial risk factors for mental disorders [46].

However, the gender disparity likely also reflects differential access to care shaped by cultural stigma. Research by Subu et al. demonstrates that Indonesian families experience profound shame regarding relatives with mental disorders, with family stigma creating barriers to seeking formal medical treatment [59]. Women with mental disorders may be more frequently hidden or managed within families due to concerns about marriage prospects, family reputation, and social standing [60, 61]. The practice of “pasung” (physical restraint) used to isolate family members experiencing mental disorders disproportionately affects women, delaying their access to medical care until symptoms become severe enough to necessitate hospitalisation [60, 61].

The concentration of hospitalisations among productive-age individuals (30–39 years) carries substantial economic implications. The Global Burden of Disease Study 2019 reported that 80.6% of the mental disorder burden occurs in individuals aged 16–65 years, with peak impact at ages 25–34 years [15]. WHO estimates that 15% of working-age adults experience mental disorders, causing global economic losses of approximately US$1 trillion annually due to decreased productivity [62]. For Indonesia, with its large working-age population, mental disorders among productive individuals significantly impact Disability-Adjusted Life Years (DALYs), labour force participation, and national productivity [15, 63, 64].

## Implications for practice and policy

### Integrated clinical pathways

Developing and implementing standardized clinical pathways is essential to reducing variability in patient outcomes and hospital costs and managing the 30-day readmission rates. The clinical practice guidelines are designed to inform decision-making and establish criteria for diagnosis, management, and treatment in hospitals. They aim to standardize medical services, enhance service quality, and reduce unnecessary interventions [65]. By providing the best treatment options, these guidelines help minimize risks to patients, healthcare providers, and insurers while achieving an optimal balance between costs and medical factors such as effectiveness, specificity, and sensitivity. Complementing these guidelines, the Integrated Clinical Pathway (ICP) offers a comprehensive service planning approach that outlines each step of patient care during hospitalisation [52]. Based on medical, nursing, and other evidence-based standards, ICPs aim to reduce service variation, making costs more predictable and care more standardized while their implementation across multiple diagnoses has shown positive impacts on patient outcomes, LOS, hospital costs, and patient satisfaction [66]. This approach not only improves the quality of care but also serves as a foundation for calculating the actual costs of treatment [67]. By improving the quality of collected information, ICPs are expected to lower costs by reducing hospital stays, while maintaining high service standards. This is particularly crucial in high-cost and high-volume cases, where ICPs provide a valuable comparison to INA-CBGs costs.

These standardized pathways are particularly crucial in managing patients with severe mental disorders, such as schizophrenia and schizoaffective disorders. Studies have shown that patients with prolonged hospitalisation times, particularly those with severe mental disorders, are at a higher risk of re-hospitalisation due to their complex clinical conditions [49, 68]. The chronic nature of these disorders, compounded by frequent comorbidities such as substance abuse and personality disorders, often results in self-injurious behaviours and poor adherence to treatment regimens [69]. Cognitive impairments, social isolation, and inadequate support systems further exacerbate these issues, leading to a revolving door phenomenon where patients are repeatedly admitted for care [70]. Therefore, implementing comprehensive treatment plans through ICPs, combined with strong support networks, is critical to improving patient outcomes and ensuring the financial sustainability of psychiatric care in Indonesia.

### Reimbursement system reform

In its health services, Indonesia has implemented a case-mix system through the INA-CBGs under the NHI programme, which is organised by the social security agency (BPJS Kesehatan). INA-CBGs are developed from the case-mix system by classifying diagnoses and procedures with the same or similar clinical characteristics and costs. The case-mix system uses tariff grouping based on diagnostic codes according to the International Classification of Diseases and Related Health Problems 10th Revision (ICD-10) [71]. As a prospective payment model, INA-CBGs determine hospital reimbursement based on fixed case-mix tariffs, regardless of the actual expenditure incurred. Under this payment structure, hospitals bear the full cost of any expenditure exceeding the fixed tariff, irrespective of clinical complexity or actual resource utilisation. Our findings provide preliminary, hypothesis-forming evidence suggesting the potential importance of investigating structural reforms to the INA-CBGs tariff system for psychiatric inpatient care, consistent with international evidence documenting the limitations of prospective payment systems when applied to clinically complex and long-stay psychiatric populations [55, 56]. As single-centre retrospective data, however, these findings do not establish the evidentiary threshold required for policy implementation. Rather, they identify specific areas where future multi-centre, longitudinal research is needed to evaluate the potential merit of reform approaches. The data are consistent with the hypothesis that the following reform directions would merit investigation: (1) development of psychiatric-specific case-mix groupings that account for the chronic, relapsing nature of severe mental disorders, in line with reform approaches adopted in several high-income OECD countries [56]; (2) implementation of outlier payment mechanisms for cases exceeding standard LOS thresholds due to documented clinical complexity, a model shown to reduce financial burden on facilities managing high-acuity patients [53, 54]; (3) adjustment of base tariffs to reflect the actual resource intensity of psychiatric care, including medication costs, therapeutic interventions, and extended nursing care requirements [27, 55]; and (4) creation of bundled payment models that incentivize quality outcomes such as reduced readmission rates, rather than throughput alone [56]. These reform directions require replication across multiple psychiatric facilities of varying levels of care, geographic regions, and ownership types before they can be considered as evidence-based inputs to national NHI policy review.

## Study strengths and limitations

This study makes several important contributions to the evidence base on psychiatric inpatient care quality within Indonesia’s NHI system. First, it is among the first studies to apply the Donabedian quality framework comprehensively to a specialised psychiatric hospital operating under the INA-CBGs prospective payment system, providing a theoretically grounded, multi-dimensional assessment of care quality that integrates clinical, financial, and structural indicators simultaneously. Second, the use of nationally administered e-claim data from BPJS Kesehatan ensures that findings reflect real-world care delivery patterns rather than curated clinical records, enhancing ecological validity. Third, the longitudinal comparison across two distinct study periods (2019 and the early pandemic period of 2020) allows examination of whether observed patterns were specific to routine conditions or persisted under disrupted service delivery. Fourth, the inclusion of unadjusted ORs, confidence intervals, and mean financial loss differentials with inferential test statistics provides quantitative characterisation of the magnitude and direction of the observed associations. Fifth, this study addresses a critical evidence gap: no prior study has rigorously assessed INA-CBGs tariff adequacy within a specialised psychiatric hospital in eastern Indonesia, a region that remains substantially underrepresented in health services research.

Several limitations should be considered when interpreting these findings. First, the single-centre design introduces institutional-level selection bias across three dimensions that constrain generalisability: (i) as the sole tertiary psychiatric referral centre for eastern Indonesia, this hospital serves the most clinically complex, treatment-refractory, and socially disadvantaged patients in the region, meaning findings should not be extrapolated to lower-level facilities without caution; (ii) the hospital’s complex case-mix dominated by severe and chronic psychiatric conditions may generate INA-CBGs reimbursement deficits substantially larger than those that would be observed at district-level or community facilities with less resource-intensive patient populations; and (iii) reimbursement adequacy patterns may differ across facility types, geographic regions, and hospital ownership status (public vs. private), none of which are captured in this study. Multi-centre studies systematically sampling facilities across these dimensions are needed to establish generalisability across Indonesia’s heterogeneous healthcare landscape. Second, the 2020 data covers only January-July, and findings from this period may reflect early COVID-19 pandemic disruptions to healthcare utilisation patterns rather than routine service delivery conditions. Third, the retrospective administrative design precludes causal inference; the associations reported should be interpreted as descriptive and hypothesis-generating rather than confirmatory. Fourth, the administrative e-claim dataset does not include validated clinical severity instruments such as the Brief Psychiatric Rating Scale (BPRS) or Positive and Negative Syndrome Scale (PANSS) precluding adjustment for illness severity and limiting the interpretability of LOS and readmission findings; future studies should link administrative data with clinical severity records to enable severity-adjusted analysis [72]. Fifth, the absence of post-discharge outpatient and community follow-up data precludes assessment of care trajectories beyond the inpatient episode, including medication adherence and community mental health service utilisation a particularly consequential gap given the high readmission rates observed. Sixth, the study cannot determine whether individual readmissions were clinically preventable; establishing preventability would require prospective clinical audit with standardized preventability criteria, which future research should incorporate to identify actionable quality improvement targets.

## Conclusion

This study demonstrates a clear misalignment between the INA-CBGs reimbursement structure and the clinical and financial demands of psychiatric inpatient services in Indonesia. The persistence of 30-day readmission rates of 7.2% (113 of 1,571 unique patients) in 2019 and 5.5% (34 of 614 unique patients) in 2020, a high proportion of prolonged hospitalisations (exceeding 43% of total admissions in both years), and significant financial losses, particularly among patients with extended stays, reveal important systemic challenges within the current prospective payment model. These challenges are compounded by service-delivery factors inherent to the care of severe and relapsing psychiatric illness, including the chronic nature of schizophrenia-spectrum disorders, the complexity of achieving clinical stabilisation within standardized timeframes, and the limited availability of post-discharge community support to prevent early readmission. Addressing these outcomes will likely necessitate a dual approach: structural reforms to ensure that INA-CBGs reimbursement adequately reflects the resource intensity of psychiatric inpatient care, alongside investments in integrated clinical pathways and community-based mental health services that extend the continuum of care beyond the inpatient episode. Both dimensions are necessary to ensure that insured patients with severe psychiatric illness receive consistent, high-quality care within a financially sustainable hospital system under Indonesia’s NHI programme.

## Data Availability

Data is provided within the manuscript.

## Abbreviations

ADHD: Attention Deficit Hyperactivity Disorder
BPJS: Badan Penyelenggara Jaminan Sosial
E-Claim: Electronic Claim
ICP: Integrated Clinical Pathways
IDR: Indonesian Rupiah
INA-CBGs: Indonesian Case-Based Groups
JKN: Jaminan Kesehatan Nasional
UHC: Universal Health Coverage
